# An experimental research into endostatin microbubble combined with focused ultrasound for anti-tumor angiogenesis in colon cancer

**DOI:** 10.1093/gastro/got038

**Published:** 2014-01-28

**Authors:** Xiufeng Zhang, Guangen Yang, Ying Zhang, Pintong Huang, Jianming Qiu, Yu Sun, Zhong Shen, Xiujun Liao, Hongsheng Xia, Shuxian Shao, Dong Wang

**Affiliations:** ^1^Department of Colorectal Surgery, The Third People’s Hospital of Hangzhou, Hangzhou City, Zhejiang, China ^2^Department of Ultrasonography, The Second Affiliated Hospital of Zhejiang University School of Medicine, Hangzhou City, Zhejiang, China ^3^The Original Biological Science and Technology Co. Ltd., Nanjing City, Jiangsu, China ^4^Department of Ultrasonography, The Third People’s Hospital of Hangzhou, Hangzhou City, Zhejiang, China

**Keywords:** colorectal cancer, tumor angiogenesis, ultrasonic cavitation, endostatin

## Abstract

**Objective:** to evaluate the therapeutic effect of targeted endostatin-loaded microbubbles, combined with improved, focused, directional ultrasound radiation for inhibition of subcutaneous translocation *in situ* colon tumor angiogenesis in colon cancer.

**Methods:** 65 BALB/c nude mice with subcutaneous translocation *in situ* colon tumors were randomly divided into five groups. Group A was the control group, without any treatments. In Group B, the mouse was treated with ultrasonic radiation. In Group C, the mouse was treated with ultrasonic radiation combined with empty SonoVue microbubbles. In Group D, the mouse was treated with ultrasonic radiation combined with empty Targestar-SA microbubbles. In Group E, the mouse was treated with ultrasonic radiation combined with endostatin microbubbles. The tumor size was measured before and 1, 14, and 28 days after irradiation. The peak intensity (PI), regional blood volume (RBV) and regional blood flow (RBF) were recorded using contrast-enhanced ultrasound. The tumor tissue was removed for pathological examination; the tumor necrosis area and microvascular density (MVD) were evaluated by immunohistochemistry.

**Results:** Tumors in Groups C, D and E were significantly smaller than in Groups A and B at 28 days after irradiation, with Group E the smallest. PI, RBF and RBV of Groups C, D, and E were significantly decreased 28 days after radiation with Group E the lowest, and significantly lower than Groups A and B (all *P* < 0.05). The tumor tissue necrosis area of Group E was clearly greater while MVD was obviously lower than the other groups (all *P* < 0.01) at 28 days after treatment.

**Conclusion:** The targeted endostatin microbubbles, combined with focused, directional ultrasound radiation can damage tumor microvasculature of subcutaneous colon translocation *in situ* colon cancer, as well as inhibit the tumor angiogenesis.

## INTRODUCTION

In 1971, Professor Folkman first proposed the theory that tumor growth depends on new angiogenesis, thus opening a new field of research [1]. The formation of new blood vessels within a tumor provides not only nutrition for tumor growth, but also channels for tumor metastasis. Once the new blood vessels grow in the tumor, the tissues with sufficient blood supply will grow rapidly and be difficult to control. The angiogenesis theory of tumor growth opens a new area of research, which in 2003 was rated as one of the world's top ten scientific and technological breakthroughs by *Science* magazine in the United States [2].

However, the anti-tumor angiogenesis methods that have been found to date nearly all use chemical or biological drugs to attack the specific biological targets of tumor angiogenesis, which not only brings hope for tumor therapy, but also places new challenges on oncologists. First, the responses to anti-tumor angiogenesis therapy vary in different types of tumor, or in different individuals with the same kind of tumor. Owing to the heterogeneity of tumors and the diversity of the angiogenesis-promoting factors released by tumors, it is impossible that any one kind of tumor angiogenesis inhibitor (TAI) can effectively restrain all types of tumors. Secondly, the tumor can release a lot of angiogenesis-promoting factors at the advanced stage but the patients could not tolerate unlimited amounts of drugs to obtain the ideal curative effect. In addition, the TAI can inhibit angiogenesis to prevent further tumor development, but has limited killing effect against the formed tumor. When tumor volume is less than 2 mm^3^, the tumor cells do not rely directly on blood supply but can obtain enough nutrition from interstitial fluid; therefore, the killing effects of TAI for this part of tumor cells are inadequate [3]. Finally, the therapeutic mechanism of anti-angiogenesis and experimental model data all support long-term medication, and the disease may return after withdrawal of drugs [4]. The mechanisms of tumor genesis and development are extremely complex; it is hard to obtain the ideal effect using only one kind of drug; therefore, at present, TAI cannot be separately applied but has to be combined with surgery, chemotherapy and radiotherapy in the comprehensive treatment of tumors.

Tumor dormancy therapy—with the purpose of suppression, embolization or blocking of tumor angiogenesis—is a mode of cancer therapy newly developed in recent years, following surgery, radiation and chemotherapy. At present, interventional vascular embolization therapy can only embolize arteries of large diameter and is powerless in the collateral circulation. The physical treatments for tumors, including thermal ablation, freezing and electrochemistry etc., can directly kill tumor tissues, but the side-effects are greater. High-intensity focused ultrasound (HIFU) treatment has rapidly progressed in recent years; the ultrasonic energy, radiated *in vitro* and focused on the target area within the body, causes the temperature of the target area to increase rapidly to above 65°C, ablating the tumor tissues through its heating effect [5]. However, because the ideal treatment acoustic window of human is less, the side-effects of HIFU thermal damage are greater, and the treatment effect is not ideal. In addition, the effect of HIFU is also not aimed at the tumor microvascular structure.

Using ultrasound for non-invasive anti-neoplastic therapy has been an aspect of ultrasonic medical research in recent years. Ultrasound-mediated microbubble destruction (UMMD) can effectively prevent tumor angiogenesis and block the tumor microcirculation, but it is difficult to prevent the recurrence of angiogenesis; at the same time, many anti-tumor angiogenesis factors have this ability. Using the existing principle of the empty lipid microbubble, we prepared a new kind of microbubble treatment for anti-tumor angiogenesis by combining the microbubble ultrasound contrast agent (SonoVue) with endostatin. After injection through the peripheral venous system, the microbubbles, combined with the modified, focused ultrasound cavitation equipment, can perform targeted irradiation of the tumor(s) of subcutaneous translocation colon cancer in nude mice, and additionally release endostatins to inhibit tumor angiogenesis, on the basis that UMMD has destroyed and blocked tumor microvasculature.

The purpose of this study was to explore a new therapeutic method of combining biological drugs and physical effects to prevent tumor angiogenesis in colon cancer.

## MATERIALS AND METHODS

### Experimental animals and grouping

This study was approved by the Research Ethics Board of The Third People’s Hospital of Hangzhou (Hangzhou, P. R. China). Experimental animals were female BALB/c nude mice aged 4–6 weeks, purchased from Yangzhou University medical center. All mice were raised and tested in the specific-pathogens-free barrier system and fed with a special granulated feed after radiation sterilization with Cobalt-60. The transfection of green fluorescent protein-expressing colon cancer HT-29 cells by subcutaneous translocation *in situ* colon tumor modelling was built by the Nanjing Yuanduan Biological Technology Co. Ltd. (Jiangsu, P. R. China), according to the method reported in literature [6].

Sixty-five mice that were successfully modeled were randomly divided into five groups of 13: Group A, the control group, in which the mice were only followed-up, without any treatment; Group B, the simple ultrasonic radiation group, in which the mice were only treated with ultrasonic radiation; Group C, in which the mice were treated with the ultrasonic radiation combined with empty SonoVue microbubbles (i.e. the commonly used clinical empty microbubbles, not carrying any medicine); Group D, in which the mice were treated with ultrasonic radiation combined with empty Targestar-SA microbubbles (this type of microbubble can carry drugs but has only been used for study purposes to date) and Group E, in which the mice were treated with the ultrasonic radiation, combined with microbubbles carrying endostatins.

### Instruments and reagents

**Figure 1.**
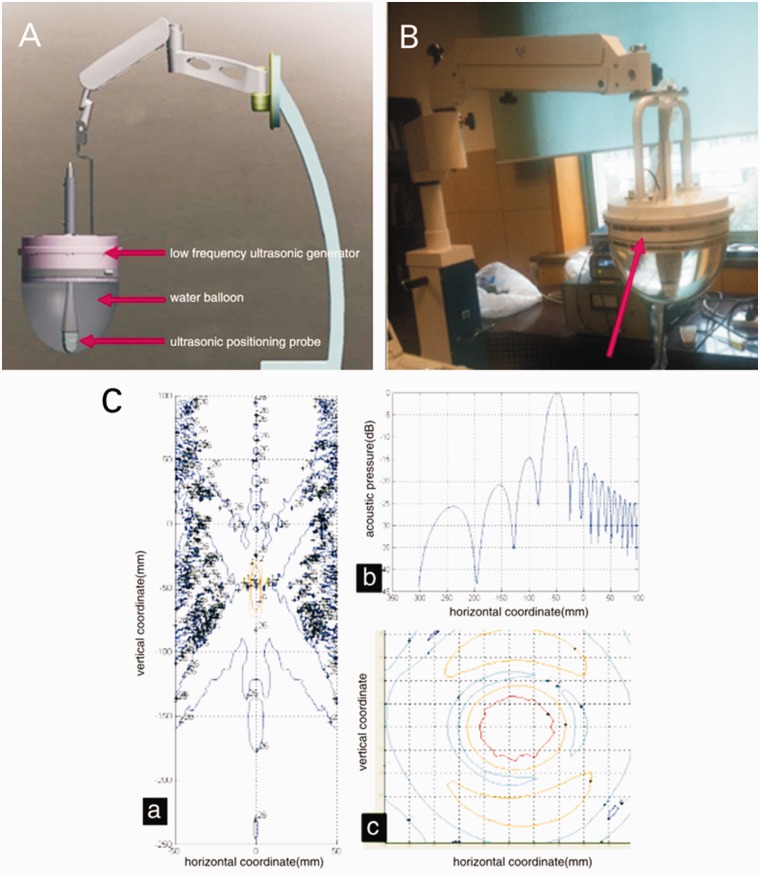
New improved cavitation therapy apparatus with focus and positioning function. (A) Simulated diagram. (B) The physical prototype, a cavitation treatment head with focus function (arrow) and built-in positioning probe. (C) The focus position and acoustic pressure distribution of this improved cavitation therapeutic apparatus, including (a) the focus position, (b) axis of acoustic pressure distribution, and (c) focal plane acoustic pressure distribution.

The modified focused impulse ultrasonic device is shown in Figure 1 (Sea Eagle Electronic Medical System Co. Ltd., Jiangsu, China). The ultrasound apparatus was a Mylab90 color Doppler ultrasonic diagnostic instrument (Esaote Co. Genova, Italy). The empty lipid microbubble Targestar-SA was supplied by Targeson Inc. (San Diego, CA, USA). The ‘empty' SonoVue contrast agent was in the form of sulfur hexafluoride microbubbles for injection (Bracco Spa., Milan, Italy). The recombinant human endostatin was Endostar, which was supplied by Shandong Simcere–Medgenn Bio- pharmaceutical Co. Ltd. (Shandong, P. R. China). Biotinylated recombinant human endostatin was compounded by Nanjing Chuanbo Biotech Co. Ltd. (Jiangsu, P. R. China). The rabbit anti-rat CD34 monoclonal antibody was supplied by Abcam plc. (Cambridge, MA, USA; Clone number: EP373Y). The IFLUOR-100 small animals living fluorescence imaging system, including the DT2000IF image analysis software, was provided by Nanjing East Colorway Digital Technology Co. Ltd. (Nanjing, P. R. China).

### Experimental methods

All nude mice models underwent fluorescence radiography examination before the trial (at 41 days after orthotopic transplantation), and images were stored. The tumor size was measured by using Image-Pro-plus v5.1 analysis software (Media Cybernetics Inc., Rockville, MD, USA), and the tumor area was calculated and recorded according to the fluorescence range, which was drawn along the edge of the fluorescence enhancement envelope curve. After completion of radiography, the configured ketamine compound anesthetic was injected intramuscularly with a 2 μL/g dose for each mouse. After the anesthesic had taken effect, the ultrasonic coupling agent was coated on the tumor surface, and the conventional ultrasonic exam was performed for tumor location. Firstly, in order to determine the best aspect, it was converted to the contrast-tuned imaging model, and the mechanical index was 0.04. Then, the drug-carrying microbubble contrast agent was slowly injected through the postocular veins with a 3.5 μL/g dose at the outer third of the infraorbital margin and pushed in vertically 5 mm or so. Meanwhile, the perfusion of contrast agents within the tumor was synchronously timed and observed in real time.

The modified focused ultrasonic broken device was started 10–20 s after the injection of contrast agent and the irradiation was performed on the tumors of Groups B, C, D, and E under real-time ultrasonic monitoring. Its working frequency was 238 kHz; the output voltage of the signal source was 400 mV, and the focus incentive sound pressure was 1.4 MPa. In total, irradiation was performed 10 times, for 10 s on each occasion, with an interval of 10 s between applications. The same irradiation process was repeated on three consecutive days (day 1, day 2, and day 3). Mice were withdrawn for feeding after the treatment, underwent the fluorescent radiography examination each week, and all images were stored for analysis. Mice were euthanized at 28 days after irradiation. Tumor tissues were then separated and excised for pathological examination and CD34 immunohistochemical and electron microscope observations.

### Observation projects

#### Fluorescence radiography image analysis

Representative fluorescence images were obtained under the fluorescence imaging system. The maximum horizontal (a) and vertical (b) diameters of the transplanted tumors were measured using the Image-Pro-plus v5.1 analysis software (Media Cybernetics Inc., Rockville, MD, USA). The volumes of tumors were calculated according to the formula v = 0.5 × a × b^2^ and then the tumor growth curve was drawn.

#### Acoustic quantitative analysis

The corresponding peak intensity (PI), regional blood volume (RBV), regional blood flow (RBF), and other imaging parameters were calculated and analysed off-line using Parkson Imaging Analysis Software Qontrast v4.0 (Italy ESAOTE S.p.A). The particular area of interest was drawn as far as possible to include the entire tumor on imaging fitting chart.

#### Pathological examination

The tumor specimen was completely excised after the mice were euthanized, fixed with 10% neutral formaldehyde, embedded in paraffin, prepared into serial sections of 4 μm thickness after conventional dehydration, and then observed for pathological changes after hematoxylin and eosin staining. CD34 immunohistochemical staining was carried out in accordance with the product manual. Pathological diagnoses were independently evaluated by two pathologists, and immunohistochemical results were then decided after discussion, by using the known positive slices of human angiosarcoma tissue as a positive control and phosphate-buffered saline, instead of primary antibodies, as a negative control.

With respect to the necrosis area (NA) of tumor tissue, the tumor necrosis region and normal cell region were observed under low-power microscope (40× magnification). The panorama of slices was scanned and the NA ratio was then calculated using the Image-Pro-plus v5.1 analysis software (Media Cybernetics Inc., Rockville, MD, USA), according to the formula NA % = region area of death cell/ total area of slice × 100.

The counting method for microvascular density (MVD) marked by CD34 was according to the Weidner standard [7]: first identify the regions of peak vascular density (hot-spots) by observing the whole slice under a low magnification (40×), then count the number of blood vessels of the five highest vascular density areas under a higher magnification (200×) and take the average value as MVD values. Single- or clusters of dyed brown endothelial cell(s) were calculated as a microvascular count, as long as they were separated from adjacent capillaries when counting, while blood vessels with diameters >8 red blood cells or with a thick muscular layer were not counted.

### Statistical analysis

All statistical analyses were performed by using SPSS v17.0 statistical software package (SPSS Inc., Chicago, IL, USA). All data were presented as the mean values ± standard deviation. Kolmogorov-Smirnov and Levene tests were used for testing normality and homogeneity of variance. Single-factor analysis of variance (one-way ANOVA) was adopted for comparisons of the means of multiple samples, and Fisher’s least significant difference test was used for the paired comparisons of multiple sample means. Paired *t*-test was used for the comparison of two sample means before and after treatment. Statistical significance was defined as a *P*-value less than 0.05.

## RESULTS

### Gross tumor volume

**Figure 2.**
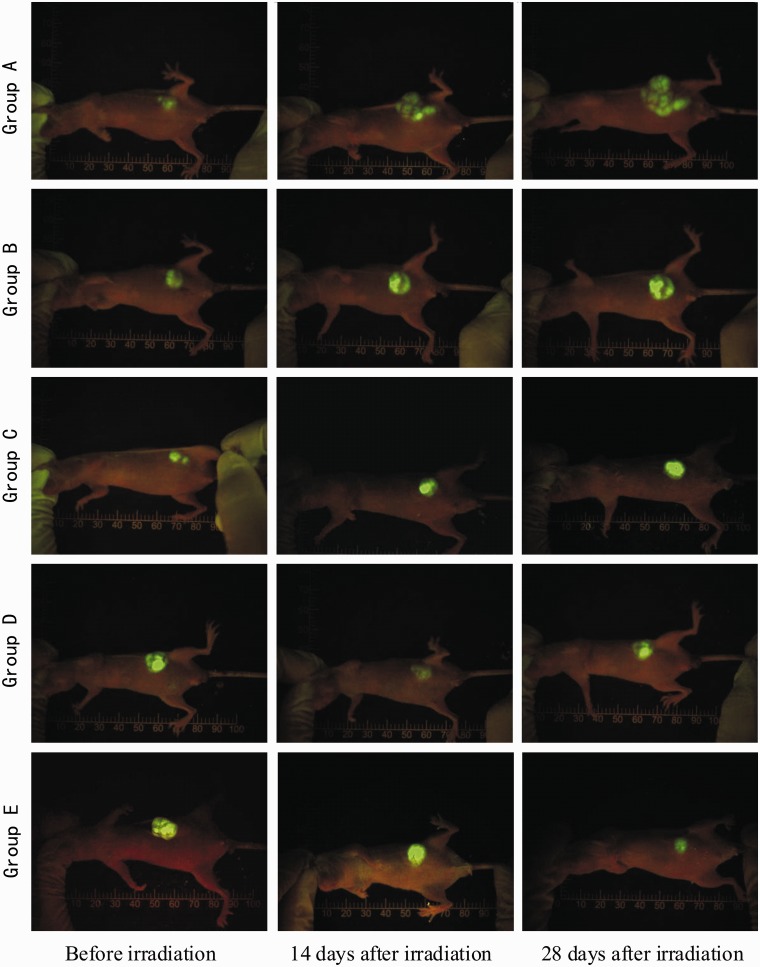
The tumor tissue of the five groups of nude mice presented visible green fluorescence on whole-body fluorescent radiography.

**Figure 3.**
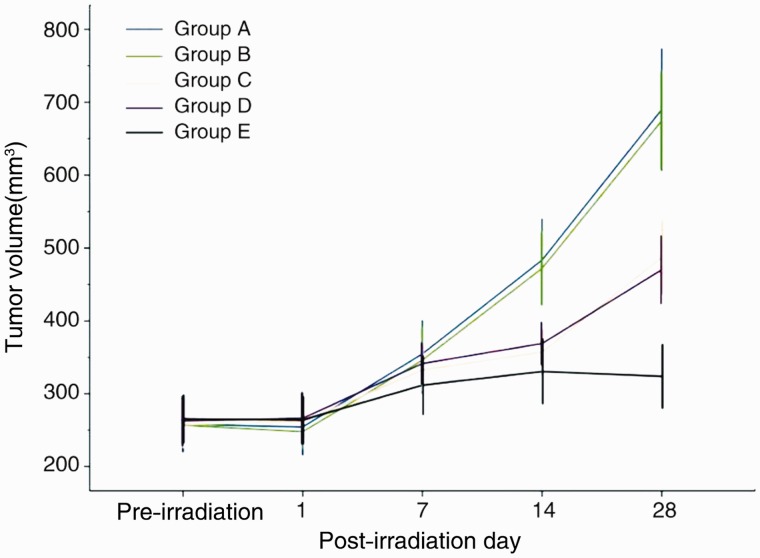
Tumor growth curves of each group nude mice.

The surface skins of each group of nude mice were intact after irradiation, without being obviously red, swollen, or burst. One mouse in Group C died from intolerance of anesthesia at 2 days after irradiation, and another in Group B unaccountably died 7 days after irradiation. Tumor fluorescence images before and after radiation for each group of mice are shown in Figure 2. There was no statistical difference in tumor size between each group (*P* > 0.05) before radiation. The tumor volumes in Groups C, D, and E were significantly lower than those of Groups A and B at 14 and 28 days after irradiation and the tumor volume of Group E was significantly lower than those of Groups A and B at 28 days after irradiation (all *P* < 0.01) (Figure 3 and Table 1).

**Table 1.** Comparisons of tumor size by group, before and after radiation [(x ± s) mm^3^]

Group	Number	Before radiation	1 day after radiation	7 days after radiation	14 days after radiation	28 days after radiation
A	13	258.0 ± 62.6	254.4 ± 62.5	354.7 ± 74.8	483.5 ± 92.1	690.2 ± 137.1
B	12	256.8 ± 44.5	247.9 ± 37.2	346.2 ± 74.6	472.1 ± 83.4	674.9 ± 112.0
C	12	257.5 ± 51.4	261.6 ± 45.4	332.6 ± 48.1	357.1 ± 50.7	487.2 ± 82.8
D	13	262.7 ± 55.6	266.3 ± 58.3	341.5 ± 46.0	368.9 ± 47.6	470.2 ± 76.2
E	13	265.4 ± 53.2	264.0 ± 52.1	311.7 ± 65.7	330.5 ± 72.4	323.9 ± 71.4
*F*-value	-	0.06	0.28	0.88	12.60	31.30
*P*-value	-	0.99	0.89	0.48	<0.01	<0.01

### Contrast-enhanced ultrasound parameters

PI, RBV, and RBF ultrasonic imaging characteristics for each group of nude mice, before and after radiation, are shown in Table 2. There were no statistical differences in PI, RBV and RBF between the five groups before irradiation (all *P* > 0.05). As compared with those before radiation, there were no statistical differences in the parameters of Groups A and B at 1 day after irradiation, (all *P* > 0.05), but there were significantly decreased parameters in Groups C, D, and E, (all *P* < 0.05). The parameters of Groups A and B significantly increased at 14 and 28 days after irradiation, compared with those before radiation, while those of Groups C, D, and E continued decreasing (all *P* < 0.05). Ultrasonic imaging parameters of Group E showed no statistical difference compared with those of Groups C and D at 14 days after irradiation (*P* > 0.05), but were significantly lower than those of Groups C and D at 28 days after irradiation (*P* < 0.05).

**Table 2.** Comparisons of ultrasonic imaging parameters before and after ultrasonic radiation (x ± s)

Group	Number	Before radiation			1 day after radiation		
		PI	RBV	RBF	PI	RBV	RBF
A	13	41.1 ± 7.0	446.9 ± 89.3	51.2 ± 7.0	42.5 ± 7.8	465.2 ± 115.9	52.5 ± 7.5
B	12	39.6 ± 6.6	418.8 ± 74.5	54.0 ± 6.7	41.3 ± 6.1	426.3 ± 57.4	52.4 ± 6.4
C	12	40.9 ± 9.5	439.6 ± 108.5	50.5 ± 6.6	36.4 ± 5.4	404.5 ± 88.9	46.1 ± 7.7
D	13	40.7 ± 7.5	431.4 ± 111.7	51.2 ± 6.9	35.2 ± 4.5	413.2 ± 106.8	45.0 ± 4.5
E	13	43.3 ± 8.6	384.8 ± 113.6	53.1 ± 7.9	39.5 ± 5.9	361.4 ± 109.9	47.8 ± 8.1
*F*-value	-	0.38	0.77	0.58	3.50	1.90	3.33
*P*-value	-	0.82	0.55	0.68	0.01	0.12	0.02

Group	Number	14 days after radiation			28 days after radiation		
		PI	RBV	RBF	PI	RBV	RBF
A	13	45.8 ± 6.9	496.8 ± 87.5	55.2 ± 6.7	47.6 ± 8.2	502.9 ± 75.8	59.7 ± 6.0
B	12	43.9 ± 6.4	465.7 ± 59.6	55.3 ± 6.0	48.1 ± 5.7	485.4 ± 55.9	61.1 ± 4.8
C	12	34.2 ± 6.3	381.6 ± 85.5	41.3 ± 5.3	31.8 ± 4.0	354.2 ± 78.1	38.2 ± 5.0
D	13	32.0 ± 4.8	396.9 ± 103.4	41.5 ± 5.1	29.3 ± 3.4	375.5 ± 98.3	38.9 ± 3.8
E	13	37.4 ± 6.1	345.1 ± 103.0	43.4 ± 5.9	27.1 ± 3.2	325.2 ± 94.8	34.9 ± 4.7
*F*-value	-	12.31	6.35	20.13	49.71	12.47	87.74
*P*-value	-	0.01	<0.01	<0.01	<0.01	<0.01	<0.01

PI = peak intensity; RBF = regional blood volume; RBV = regional blood flow.

### Pathological results

**Figure 4.**
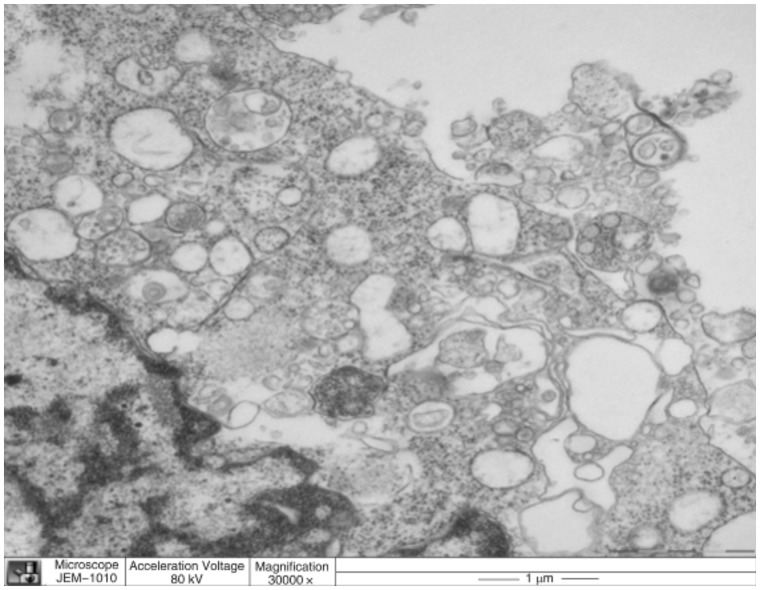
Electron microscopy images of nude mice orthotopic colon cancer treated with targeted endostatin microbubble combined with ultrasonic radiation.

**Figure 5.**
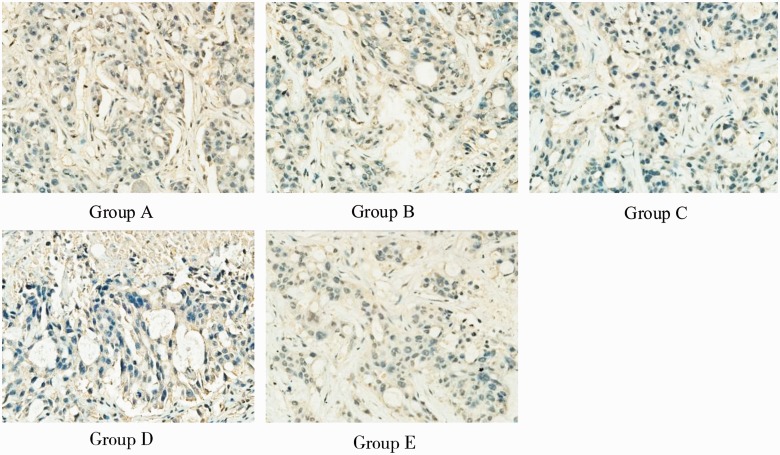
CD34 immunohistochemical staining images of nude mice *in situ* colon cancer for the five groups of nude mice at 28 days after radiation (200× magnification).

The paraffin sections of tumor tissue, after hematoxylin and eosin staining, revealed rupture of capillaries in endothelial cells, widened endothelial gap, tumor cell necrosis, and cavitation. Electron microscopy showed that the tumor cell nuclear membrane disappeared in Groups C, D, and E, nuclear chromatin dissolved and clustered in an irregular arrangement, and injury and bleeding in mitochondria, vacuoles and microvascular endothelial cells, especially in Group E, while this was rare in Groups A and B (Figure 4). Immunohistochemical staining showed that CD34 positively expressed at the endothelial cell membrane and cytoplasm, as tan particles (Figure 5). There were statistical differences in tumor tissue NA and MVD between the five groups at 28 days after treatment (*P* < 0.01): the tumor tissue NA and MVD of Group E were significantly higher and lower, respectively, than those of the other groups (Table 3).

**Table 3.** Comparison between NA within tumors and MVD for each group of nude mice at 28 days after radiation

Groups	Number	NA within tumor (%)	MVD (x ± s)
A	13	17.7 ± 3.6	23.0 ± 4.5
B	12	16.9 ± 3.5	22.6 ± 4.1
C	12	23.5 ± 4.4	21.0 ± 3.7
D	13	24.4 ± 4.7	19.6 ± 3.5
E	13	27.3 ± 5.7	17.5 ± 3.2
*F*-value	-	13.10	4.59
*P*-value	-	<0.01	<0.01

NA = necrosis area; MVD = microvascular density.

## DISCUSSION

Tumor growth depends on angiogenesis. The simple diffusion process cannot guarantee the oxygen and nutrition supply needed for growth when tumor tissue volume is greater than 2 mm^3^ or tumor cell numbers are greater than 10^7^. The tumors must build their own blood vessel systems to maintain continuous growth and transfer. If new blood vessels cannot grow, tumor tissues will remain in a dormant state or degradation will occur [1]. Tumor angiogenesis is stimulated by a variety of vascular growth factors, with rapid growth and maintenance of the original structure. Tumor angiogenesis embodies congenital developmental defects—such as weak vessel walls, incomplete basal membrane, high permeability and lack of an elastic fiber layer—and specific receptor targets for vascular endothelial growth factor (VEGF) [8]. Therefore, the anti-tumor angiogenesis therapy targets mainly the VEGF signal transduction pathways. In 1997, O’Reilly *et al.* first discovered endostatin [9], and proved that it could inhibit tumor angiogenesis in a variety of ways, such as blocking the combination of VEGF with receptors [10]. Related to this, the tumor suppression effects of new drugs of recombinant human endostatin (Endostar) explored by our national researchers on animal testing is at least twice that of endostatin [11]. We successfully attached Endostar to empty lipid microbubbles by adopting the biotin-avidin binding method, and the detection report showed that each microbubble can combine with 80–220 × 10^3^ ligand molecules on the surface (relating to technology patents), which laid a solid foundation for targeted ultrasonic cavitation drug therapy.

Using ultrasonic energy, non-traumatic treatment for tumors is one of the new ‘hot-spots' in tumor treatment in recent years. The microbubble can produce a significant cavitation effect under ultrasonic radiation [12, 13]. With the growth of research into ultrasound–microbubble biological effects, the vascular effects of microbubble ultrasonic cavitation are receiving more and more attention. In the first instance, using the cavitation effect can disrupt microvascular and surrounding tissues that supply the tumor, whilst stimulating and enhancing the activity of coagulation enzymes, forming thromboses to block blood vessels, and cut off the tumor blood supply. Further, such an effect can damage the tumor cell membrane structure, decrease proliferation and transfer ability, and leave tumors susceptible to attack from the auto-immune system and to damage by radiation and chemotherapy, also inhibiting tumor growth [14, 15]. Ultrasonic cavitation occurs under low-level ultrasound, and the mechanical energy thus released is a non-thermal effect, which avoids the thermal ablation side-effects of high-intensity focused ultrasound. It is thus receiving increasing attention in the field of minimally invasive tumor treatment.

It has been confirmed by existing research that intravenous lipid microbubble contrast agent, combined with percutaneous pulsed, focused ultrasound radiation, can effectively disrupt the physical structure of tumor angiogenesis and block tumor microcirculation for more than 24 h in rat tumors [16]. Pathological observation confirmed that the mechanism of ultrasonic microbubble cavitation in damaging tumor blood vessels is associated mainly with the physical and mechanical effects of cavitation (such as shock waves and microjet energy), which disrupt the immature and fragile process of tumor angiogenesis, causing irregular bleeding, hematoma accompanied by thrombosis, and blocking tumor microcirculation. UMMD for blocking tumor angiogenesis is simple, effective, and has a good repeatability, which may lead to a new kind of physical anti-tumor angiogenesis therapy. Unfortunately, although UMMD can physically destroy the tumor blood vessels and is repeatable, it cannot prevent the resumption of angiogenesis stimulated by hypoxia and angiogenesis factor after 48 h; additionally, it cannot destroy emergent blood vessels that have not yet formed a lumen. In this study, we combined two methods of biological and physical treatment, injected microbubbles carrying endostatin into nude mice with subcutaneous translocation *in situ* colon tumors, and simultaneously performed the focused ultrasound irradiation. As the ultrasonic cavitation damaged and blocked the tumor capillaries, the endostatin was released and the blocking of tumor microcirculation led to time-extended drug retention, so as to bring about a dual anti-tumor effect. It was found from the tumor volume growth curves that tumor sizes in Groups C, D, and E were significantly lower than those of Groups A and B (*P* < 0.01) at 28 days after radiation, and the tumor volumes in Group E were clearly lower than those of Groups C and D (*P* < 0.01), which confirmed that the strongest effect for inhibiting tumor growth was obtained from microbubbles carrying drugs, combined with ultrasonic cavitation.

PI, RBV, and RBF are good ultrasonic imaging parameters for reflecting the blood supply and perfusion of a tumor. In this study, the PI, RBV, and RBF of Groups A and B were significantly higher after treatment than before (*P* < 0.05), while the same characteristics of Groups C, D and E gradually reduced the values of PI, RBV, and RBF after irradiation (*P* < 0.05). Results illustrated that using ultrasonic radiation alone, without combining microbubble contrast agent, has no obvious effects in damaging the tumor, while the disrupt effect of ultrasound radiation combined with microbubble contrast agent damaged and blocked the tumor vasculature, causing tumor hypoxia and decreasing the tumor blood supply. Mice in Group E were given both endostatin-loaded microbubbles and ultrasonic radiation, which resulted in PI, RBV, and RBF values significantly lower than those of the other groups (*P* < 0.05) at 28 days after radiation. This is consistent with our previous results [17] and suggests that the drug-loaded microbubbles, combined with ultrasonic cavitation treatment, can reduce the blood supply of tumors, as well as inhibit growth by destroying tumor blood vessels.

CD34 is one of the most sensitive endothelial markers, with high specificity and good repeatability, which has been used as a regular indicator for immunohistochemical detection of MVD in colorectal cancer and determination of the tumor angiogenesis [18]. In this study, the NA and MVD of Group E were higher and lower, respectively, than those of the other groups at 28 days after treatment, (all *P* < 0.01). Electron microscopy showed that the tumor cell nuclear membrane disappeared in Groups C, D, and E, nuclear chromatin dissolved and clustered in an irregular arrangement, and mitochondria vacuoles and microvascular endothelial cells were injured and bleeding. All these manifested most obviously in Group E, but rarely in Groups A and B, which suggests that ultrasonic cavitation, combined with targeted microbubbles, can enhance the damage to tumor cells tumor microvasculature, but that the effect of targeted drug-loaded microbubbles is much better. The endostatin-loaded microbubbles were better able to target, adhere to, and accumulate within the tumor, and enhance the physical effect of ultrasonic cavitation, directly damaging the tumor microvasculature. Also, as the ultrasonic cavitation damages and blocks the tumor microvasculature, the microbubbles simultaneously release the endostatin, which can effectively inhibit tumor angiogenesis and further enhance curative effects.

In this study, we adopted the subcutaneous translocation colon cancer fluorescence *in situ* transplantation nude mouse model rather than the subcutaneous colon nude mouse transplantation tumor model, which can better reflect the biological characteristics of colon tumor, and the fluorescence can be observed relatively early, to determine the tumor size, location, and whether transfer occurs. In addition, the tumor is more easily located during the ultrasonic cavitation treatment, and the therapeutic effect is much better. Our research group co-operated with the Wuxi Sea Eagle Electronic Medical System Co. Ltd., (Jiangsu, P. R. China), improving the defects and shortcomings of the original ultrasonic cavitation device. The improved cavitation therapy apparatus has a good deep focus on performance and can be operated with conventional ultrasonic positioning, with longer focal length and higher axis pressure, which can be used for the treatment tumors with further improved therapeutic effects. This study has applied for patent (Application No. 201110161278, entitled “Focusing ultrasonic cavitation treatment instrument with ultrasonic focusing positioning function"). The improvement of metrics and mechanisms of the therapeutic apparatus was just simply introduced within the article, because of involving technology patents.

There are some limitations to this study, such as failure to monitor changes of tumor tissue temperature after cavitation treatment, although the thermal effect was very weak. In addition, the sample size was limited, which was restricted by the shorter observation time of the tumor model. Therefore, further in-depth study is recommended.

In conclusion, ultrasonic cavitation combined with drug-loaded microbubbles shows good clinical prospects in the treatment of colorectal cancer.

## FUNDING

This work was supported by the following grants: National Natural Science Foundation of China (No. 81071164, No. 81271584), Zhejiang Provincial Natural Science Foundation of China (No. Y2110782), Public Technology for Applied Research Program Foundation from the Science and Technology Bureau of Zhejiang Province (No. 2013C31002), Key Program Foundation from the Health Bureau of Zhejiang Province (No. 2013ZDA020), and the General Program Foundation from the Hangzhou City Health Bureau (No.2013A27).

**Conflict of interest:** none declared
